# Effects of empagliflozin and its combination with docetaxel on LNCaP and DU- 145 prostate cancer cell lines: cytotoxicity and molecular pathway analysis

**DOI:** 10.1007/s00210-025-04132-9

**Published:** 2025-04-14

**Authors:** Ahmet Cumaoğlu, Selda Nur Akdeniz, Ahmad Karzoon, Mohamed Alaama, Mükerrem Betül Yerer

**Affiliations:** 1 Department of Biochemistry, Faculty of Pharmacy, Erciyes University, Kayseri, Türkiye; 2 Department of Pharmacology, Faculty of Medicine, Erciyes University, Kayseri, Türkiye; 3 Drug Application and Research Center (ERFARMA), Biopep Medikal İlaç Sanayi Ve Ticaret, Erciyes University, Kayseri, Türkiye; 4 Department of Pharmacology, Faculty of Pharmacy, Erciyes University, Kayseri, Türkiye

**Keywords:** Empagliflozin, Prostate cancer, AMPKα, PGC- 1α, PRAS40, P70S6 K1

## Abstract

Sodium-glucose cotransporter 2 (SGLT2) inhibitors have gained attention for their potential therapeutic applications in cancer treatment. Recent Mendelian randomization and observational analyses support the protective role of SGLT2 inhibition in reducing prostate cancer risk. Additionally, SGLT2 expression in prostate cancer patient samples has been confirmed through immunohistochemistry. The therapeutic potential of empagliflozin, a selective SGLT2 inhibitor, in treating prostate cancer, either alone or with chemotherapeutic agents like docetaxel, remains largely unexplored. This study investigated the cytotoxic and synergistic effects of empagliflozin in combination with docetaxel in LNCaP and DU- 145 prostate cancer cells. Cell viability was assessed using the MTT assay, and synergy was evaluated using the Chou-Talalay method. Western blot analysis was conducted to examine the effects of empagliflozin, alone and in combination with docetaxel, on key molecular targets, including p-AMPKα, p-p70S6 K1, p-PRAS40, and p-Akt. Empagliflozin exhibited concentration-dependent cytotoxic effects in both LNCaP and DU- 145 cells, with a higher potency observed in DU- 145 cells. When combined with docetaxel, empagliflozin demonstrated synergistic activity, as indicated by combination index values < 1. Empagliflozin upregulated p-AMPKα and downregulated p-p70S6 K1 and p-PRAS40. The combination with docetaxel further enhanced these effects. Notably, empagliflozin alone downregulated p-Akt in LNCaP cells but not in DU- 145 cells, highlighting cell-line-specific differences. Empagliflozin reduces prostate cancer cell viability and enhances the cytotoxic effects of docetaxel, suggesting a promising combination strategy for prostate cancer therapy. Additional in vivo studies and clinical trials are needed to assess the translational relevance of these findings.

**Supplementary Information:**

The online version contains supplementary material available at 10.1007/s00210-025-04132-9.

## Introduction

The widespread occurrence of prostate cancer and its related mortality pose substantial challenges to healthcare systems worldwide. In 2022, prostate cancer accounted for over 1.46 million newly diagnosed cases and 396,792 deaths globally (Bray et al. 2024). If current trends continue, the burden of prostate cancer is projected to rise significantly, with estimates exceeding 2.17 million new cases and more than 0.72 million deaths by 2040 (Kalita et al. 2024).

The standard chemotherapy for prostate cancer is primarily based on docetaxel, while hormonal therapy remains a cornerstone of treatment. While nearly all patients initially respond to hormonal therapy, the duration of this response varies, ranging from months to years, before the disease inevitably progresses to its lethal stage, known as castration-resistant prostate cancer (CRPC). In the setting of CRPC, docetaxel serves as a key therapeutic option for patients who are refractory to androgen receptor (AR)-targeting agents. However, resistance typically develops after a median prostate-specific antigen (PSA) response duration of 7 to 8 months (Tannock et al. 2004; Watson et al. 2015). These findings highlight the need to optimize treatment strategies by integrating novel therapeutic targets and innovative combination approaches.

Zheng et al. (2024) conducted a study using Mendelian randomization and observational analysis, which provided multiple lines of evidence supporting the beneficial effect of SGLT2 inhibition in reducing prostate cancer risk. Additionally, they found little evidence linking HbA1c levels with prostate cancer. Moreover, immunohistochemical analysis confirmed SGLT2 expression in human prostate cancer samples, whereas no specific SGLT2 staining was observed in normal ducts or acini of the prostate (Scafoglio et al. 2015). Furthermore, prostate cancer tissues exhibited a 2.02-fold higher SGLT2 expression compared to adjacent normal tissues (Zheng et al. 2024).

Empagliflozin demonstrates one of the highest selectivities for SGLT2 over SGLT1 (2680:1) among other SGLT2 inhibitors. However, its therapeutic effects, both alone and in combination with docetaxel, remain largely unexplored in prostate cancer.

Empagliflozin has been shown to inhibit mTOR activity—not only by activating AMPK (Chaube et al. 2015) but also by suppressing Akt, as reported in ER-positive breast cancer cells (Karzoon et al. 2025). Similarly, our hypothesis proposes that empagliflozin increases intracellular AMP levels, which activates AMPKα. This activation subsequently suppresses p70S6 K1 (a substrate of mTORC1) and Akt. Additionally, PRAS40—a 40-kDa proline-rich substrate of Akt and a component of mTORC1—functions at the intersection of the PI3 K/Akt and mTOR pathways. Its phosphorylation, frequently linked to tumor progression in prostate cancer, causes PRAS40 to dissociate from mTORC1, thereby lifting its inhibitory influence on mTORC1 activity (Kazi and Lang 2010; Shipitsin et al. 2014).

This study investigates the anticancer effects of empagliflozin and evaluates its potential synergistic interaction with docetaxel in LNCaP and DU- 145 prostate cancer cells. We assess the impact of both empagliflozin alone and in combination with docetaxel on critical molecular targets involved in prostate cancer progression, including AMPKα, p70S6 K1, PRAS40, and Akt.

## Materials and methods

### Materials and reagents

Empagliflozin (#BD289522) was purchased from BLD Pharm (Shanghai, China). Docetaxel (#ab141248) was purchased from Abcam (Cambridge, UK). Advanced Roswell Park Memorial Institute (RPMI)− 1640 (#12633020), Dulbecco's phosphate buffered saline (#14190–094), and trypsin–EDTA (#25200–056) were purchased from Life Technologies, Inc. (Paisley, UK). Fetal bovine serum (#F9665) and penicillin–streptomycin (#P4333) were purchased from Sigma-Aldrich (St. Louis. MO, USA). All the other chemicals utilized were of analytical grade.

### Cell culture

LNCaP and DU- 145 prostate cancer cell lines were obtained from American Type Culture Collection. Both were cultured in RPMI- 1640 supplemented with 10% fetal bovine serum (FBS) and 1% penicillin–streptomycin. All cultures were incubated at 37 °C in a humidified atmosphere with 5% CO₂.

### Cell viability assay

LNCaP and DU- 145 cells were plated at 8,000 cells per well in RPMI- 1640 containing 10% FBS in a 96-well plate for 24 h. They were treated with empagliflozin at different concentrations (75–600 µM), docetaxel (1.25–80 µM) or combinations of both for 48 h. Then at the end of the incubation period, cell viability was measured using MTT assay. A 5 mg/ml of the tetrazolium salt, 3-(4,5-dimethylthiazolyl- 2)− 2,5-diphenyltetrazolium bromide (MTT) solution was added to each well and the plates were incubated for an additional 45 min at 37 °C. Optical density measurements were carried out using a multimode microplate reader (SpectraMax® Molecular Devices) at 562 nm. Cell viability is expressed as a percentage of the mean of the untreated control. The values of IC_50_ were calculated from concentration–response curves.

### Protein extraction and western blot analysis

LNCaP and Du- 145 Cells were seeded in T25 flasks. Upon reaching 70–75% confluency, LNCaP cells were treated with 380 μM empagliflozin, 18 μM docetaxel, or a combination of both, while DU- 145 cells were treated with 260 μM empagliflozin, 16 μM docetaxel, or a combination of both. Control groups received vehicle treatment. Cells were collected at two time points: 24 and 48 h of treatment. After treatment, cells harvested in RIPA lysis buffer containing protease and phosphatase inhibitors and then centrifuged for 15 min at 12,000 rpm. Protein concentrations were determined using the BCA Protein Assay Kit (ABP Biosciences, Maryland, USA). For each sample, 30 μg of protein was loaded into a 10% SDS–polyacrylamide gel. Protein was transferred into PVDF membranes (Nepenthe, Türkiye) using trans-blot turbo transfer system from Biorad™. Nonspecific binding sites on the membranes were blocked with 5% bovine serum albumin in TBS-T. Following blocking, the membranes were incubated overnight at 4 °C with the following primary antibodies: phospho Thr172-AMPKα (#2535; Cell Signaling Technology), phospho Ser371 p70S6 K1 (#9208, Cell Signaling Technology), phospho Ser473-Akt (#AF0016, Affinity Biosciences), phospho Thr246-PRAS40 (#AF2387, Affinity Biosciences) and GAPDH (#E-AB- 40337, Elabscience). After washing, the membranes were incubated with goat anti-rabbit IgG secondary antibodies conjugated with horseradish peroxidase (#A53211, AFG Bioscience) in TBS-T for 2 h at 4 °C. The experiments were conducted in triplicate, and signals were detected using an enhanced chemiluminescence (ECL) kit (#SM801 - 0500, GeneDireX Inc., Taiwan) and the ChemiDoc XRS Imaging System. The bands were analyzed using ImageJ software (National Institutes of Health, USA).

### Statistical analysis

The results are expressed as the mean ± standard deviation (SD). Statistical evaluation was performed using one-way analysis of variance (ANOVA) with a subsequent Bonferroni post-test. Statistical significance was established at *P* < 0.05. All analyses were carried out using GraphPad Prism (GraphPad Software Inc., San Diego, CA, USA).

## Results

### Empagliflozin exhibits cytotoxic effects and demonstrates synergistic activity with docetaxel in LNCaP and DU- 145 prostate cancer cell lines

**Fig. 1:**
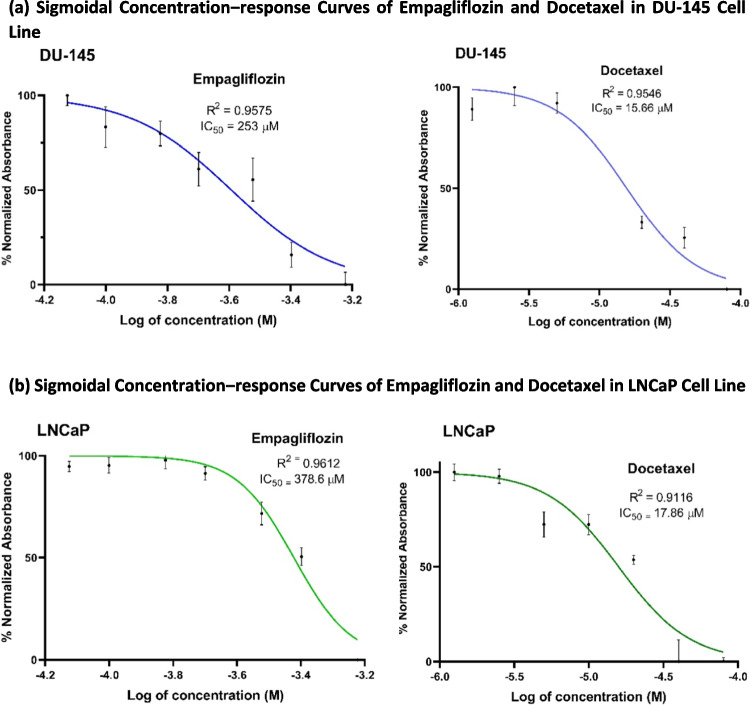

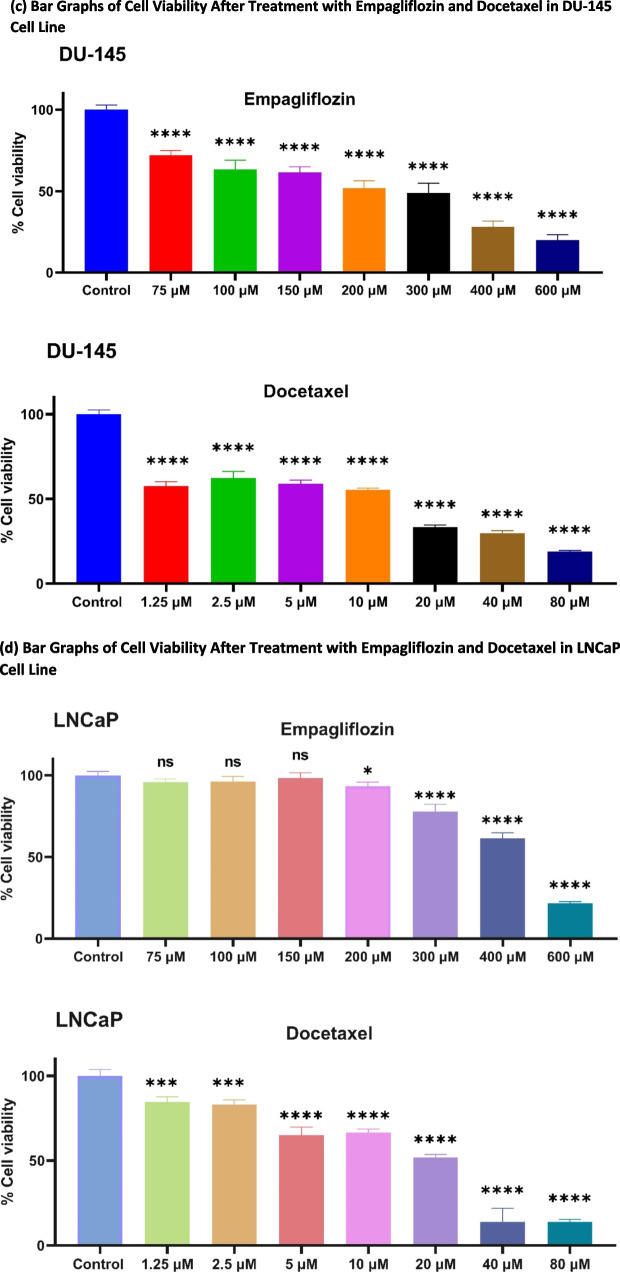
Cytotoxic effects of empagliflozin and docetaxel individually in LNCaP and DU- 145 prostate cancer cell lines. Cells were treated with varying concentrations of empagliflozin (75 to 600 μM) or docetaxel (1.25 to 80 μM) for 48 h. Figures (**a**) and (**b**) illustrate sigmoidal concentration‒response curves. Figures (**c**) and (**d**) represent bar graphs of cell viability. Cell viability is expressed as a percentage of the mean of the untreated control. Both empagliflozin and docetaxel demonstrated concentration-dependent cytotoxic effects. The data are presented as the means ± standard deviations (SDs), and each experiment was performed in triplicate (*n* = 3). Statistical significance levels are indicated as * *p* < 0.05, *** *p* < 0.001, and **** *p* < 0.0001 when compared with the control, while"ns"indicates non-significance

Initially, we evaluated the cytotoxic effects of empagliflozin and docetaxel individually in LNCaP and DU- 145 prostate cancer cells using the MTT assay. The IC_50_ values for both drugs were determined after 48 h of treatment. As shown in Fig. 1, both drugs exhibited concentration-dependent cytotoxicity, with docetaxel demonstrating significantly higher potency. In LNCaP cells, docetaxel was approximately 21 times more potent (IC_50_ = 17.86 μM) than empagliflozin (IC_50_ = 378.6 μM), while in DU- 145 cells, it was about 16 times more potent (IC_50_ = 15.66 μM vs. 253 μM). Notably, empagliflozin induced significant cytotoxicity at 200 μM, whereas all tested concentrations of docetaxel showed significant cytotoxicity in LNCaP cells. DU- 145 cells displayed greater sensitivity to both drugs, with significant reductions in cell viability observed at 75 μM for empagliflozin and at 1.25 μM for docetaxel, where the cytotoxic effect was more pronounced compared to LNCaP cells.

Our next investigation sought to determine whether empagliflozin exhibits synergistic effects when combined with docetaxel. LNCaP and DU- 145 prostate cancer cells were treated with a combination of empagliflozin and docetaxel at equipotent ratios of 21:1 for LNCaP and 16:1 for DU- 145 cells. These ratios were based on docetaxel’s approximately 21-fold and 16-fold greater potency than empagliflozin in LNCaP and DU- 145 cells, respectively (see Fig. 1a and b). Additionally, cells were treated with a fixed concentration of empagliflozin combined with increasing concentrations of docetaxel, considering the enhanced sensitivity of the cells to incremental increases in docetaxel levels.

To evaluate potential synergistic effects, combination index (CI) values were calculated using the Chou-Talalay method (Chou 2010) via CompuSyn software, with CI values less than 1 indicating synergism, equal to 1 suggesting additivity, and greater than 1 reflecting antagonism.

**Fig. 2:**
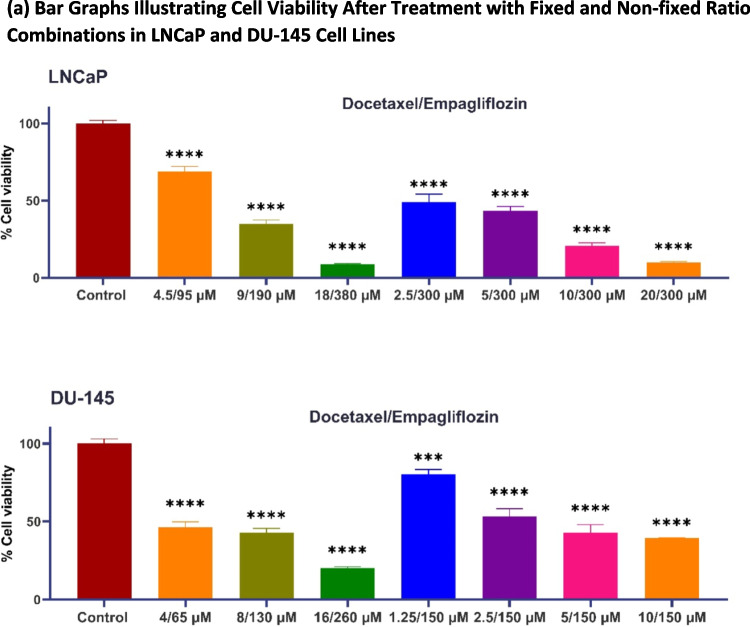

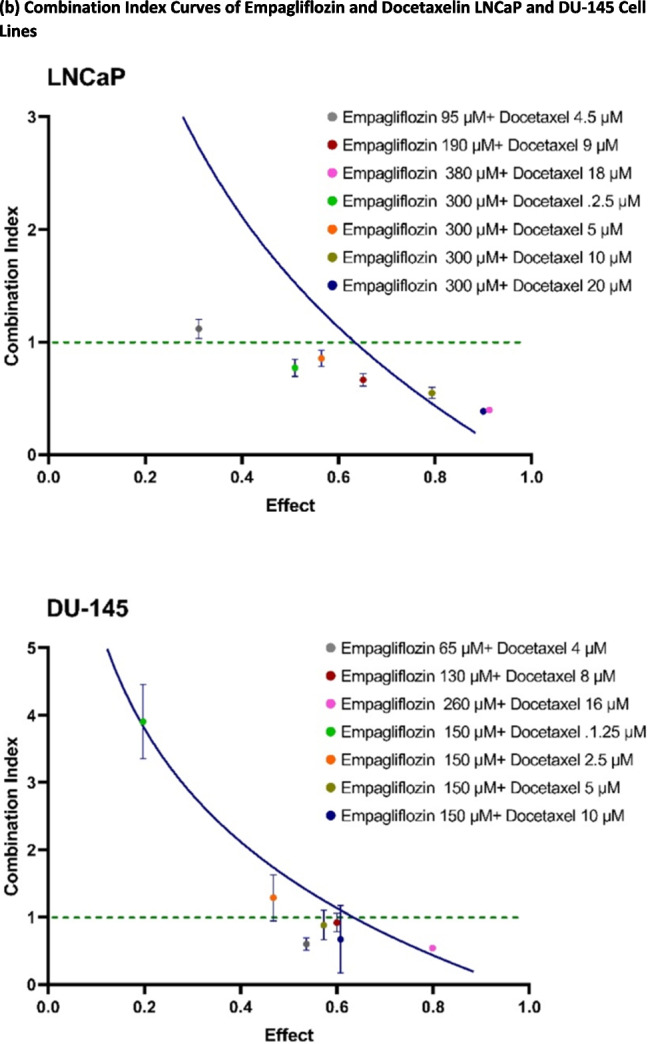
Synergistic cytotoxicity of empagliflozin and docetaxel across various combination regimens in LNCaP and DU- 145 cells. Figure (**a**) displays bar graphs representing cell viability following 48 h of concurrent treatment with empagliflozin and docetaxel in LNCaP and DU- 145 prostate cancer cells. Viability is expressed as a percentage relative to the untreated control. Figure (**b**) illustrates the combination index (CI) plots for different combination regimens of empagliflozin and docetaxel, where values below and above the dashed line (CI = 1) indicate synergistic and antagonistic interactions, respectively. The data are presented as the means ± standard deviations (SDs) (*n* = 3), with statistical significance levels indicated as *** *p* < 0.001 and **** p < 0.0001 when compared with the control

The combination index plots (Fig. 2b) demonstrate a general decrease in CI values as the effect level increases. In LNCaP cells, treatment with 300 µM empagliflozin alongside docetaxel doses ranging from 2.5 µM to 20 µM consistently resulted in CI values below 1, indicating a synergistic interaction. The strongest synergy was observed with 300 µM empagliflozin and 20 µM docetaxel, yielding the lowest CI value. Conversely, combinations involving lower concentrations of empagliflozin (e.g., 95 µM) and docetaxel (e.g., 4.5 µM) showed CI values slightly above 1, suggesting a minor shift toward antagonism at lower efficacy levels. In DU- 145 cells, all tested combinations except for 150 µM empagliflozin with docetaxel doses between 1.25 µM and 2.5 µM yielded CI values below 1, supporting a synergistic effect across these doses. Overall, these results highlight a dose-dependent synergistic mechanism between empagliflozin and docetaxel in LNCaP and DU- 145 prostate cancer cells, with the strongest synergistic effects occurring at higher drug concentrations.

Additionally, we calculated dose-reduction index (DRI) values utilizing CompuSyn software. The DRI quantifies the fold decrease in dose achievable through a synergistic combination at a specific effect level, relative to the dose required when each drug is used independently. Tables 1 and 2, corresponding to LNCaP and DU- 145 cell lines, respectively, reveal that the DRIs for both empagliflozin and docetaxel exceed 1, suggesting a beneficial dose reduction when these agents are used in combination (Chou 2010).

**Table 1:** Dose reduction ındices (DRIs) of Empagliflozin and Docetaxel in LNCaP cell line

Combination	Dose reduction index (DRI)	
	Empagliflozin	Docetaxel
95 μM Empagliflozin + 4.5 μM Docetaxel	3.61	1.10
190 μM Empagliflozin + 9 μM Docetaxel	3.40	2.56
380 μM Empagliflozin + 18 μM Docetaxel	3.66	8.24
300 μM Empagliflozin + 2.5 μM Docetaxel	1.66	4.91
300 μM Empagliflozin + 5 μM Docetaxel	1.83	3.11
300 μM Empagliflozin + 10 μM Docetaxel	2.97	5.04
300 μM Empagliflozin + 20 μM Docetaxel	4.34	6.31

**Table 2:** Dose reduction ındices (DRIs) of Empagliflozin and Docetaxel in DU- 145 cell line

Combination	Dose reduction index (DRI)	
	Empagliflozin	Docetaxel
65 μM Empagliflozin + 4 μM Docetaxel	3.51	2.78
130 μM Empagliflozin + 8 μM Docetaxel	1.99	1.76
260 μM Empagliflozin + 16 μM Docetaxel	2.66	5.76
150 μM Empagliflozin + 1.25 μM Docetaxel	0.37	0.62
150 μM Empagliflozin + 2.5 μM Docetaxel	1.19	2.77
150 μM Empagliflozin + 5 μM Docetaxel	1.73	2.85
150 μM Empagliflozin + 10 μM Docetaxel	1.97	1.83

Based on the observed individual and combined cytotoxic effects, we selected the combination of 380 µM empagliflozin with 18 µM docetaxel for subsequent assays in LNCaP cells, and the combination of 260 µM empagliflozin with 16 µM docetaxel for further experiments in DU- 145 cells.

### Empagliflozin and its combination with docetaxel upregulate p-AMPKα and downregulate p-p70S6 K1 and p-PRAS40, with p-Akt downregulation in LNCaP cells and no effect in DU- 145 cells

The effects of docetaxel, empagliflozin, and their combinations on p-AMPKα, p70S6 K1, p-Akt, and p-PRAS40 levels were assessed in DU- 145 and LNCaP cell lines at 24 and 48 h using Western blotting.

**Fig. 3:**
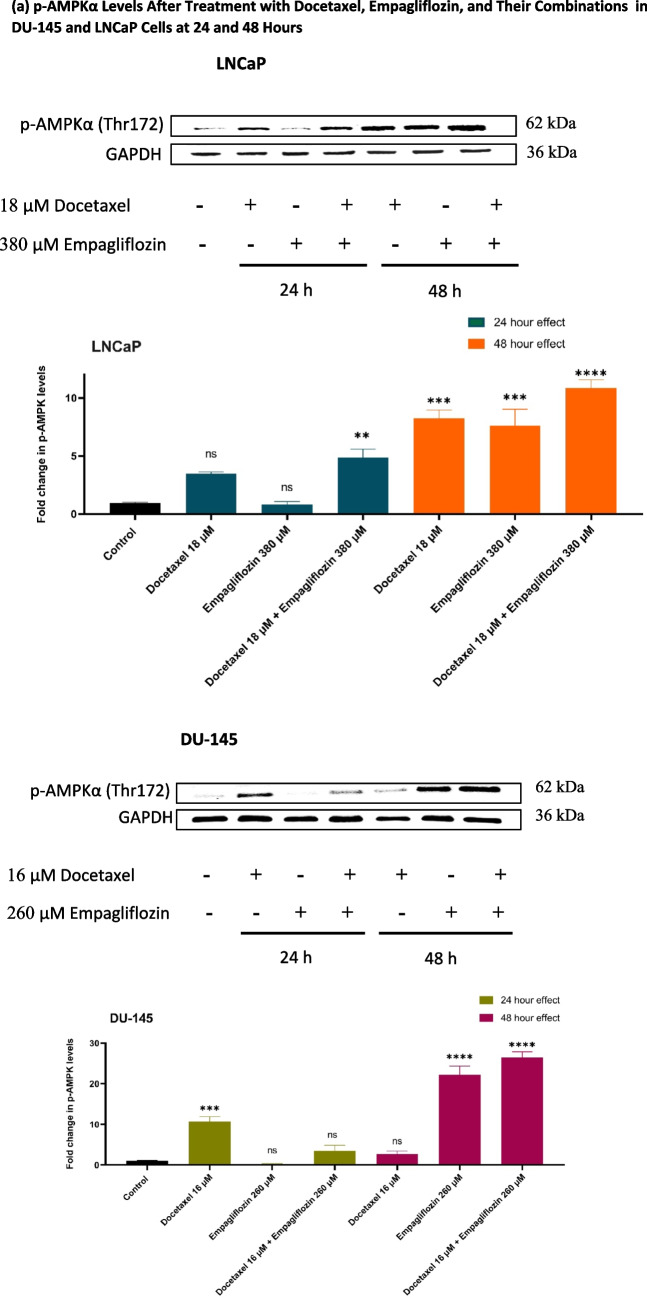

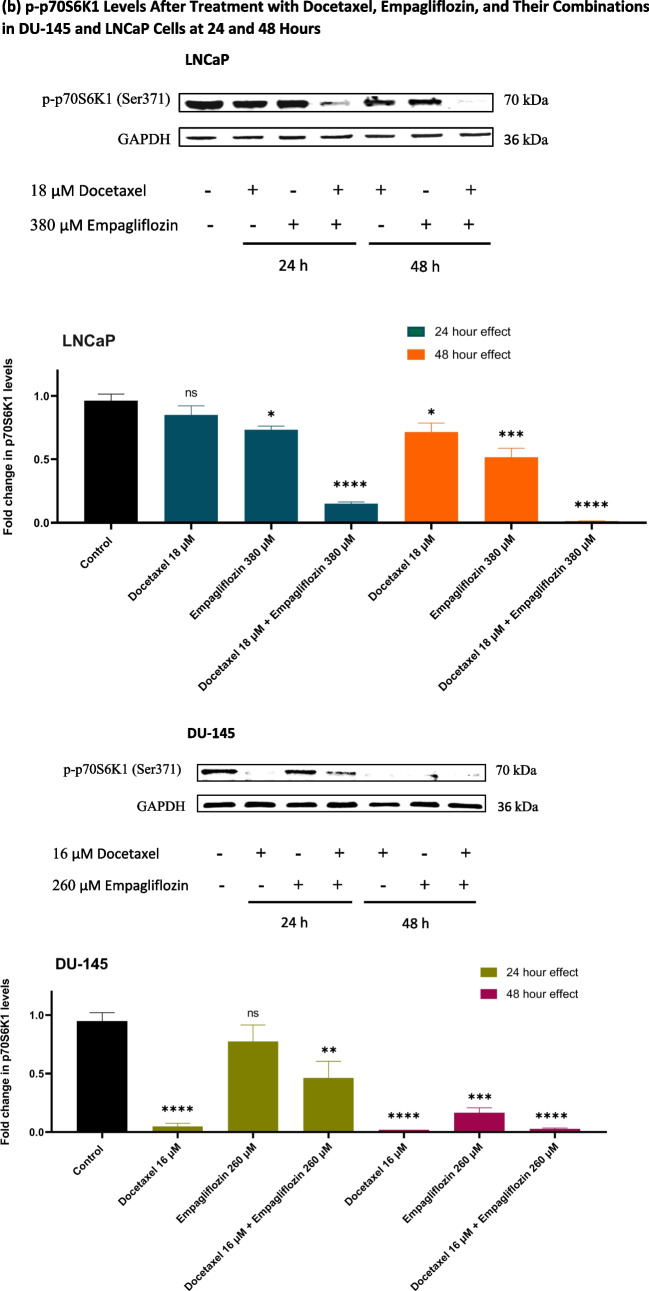

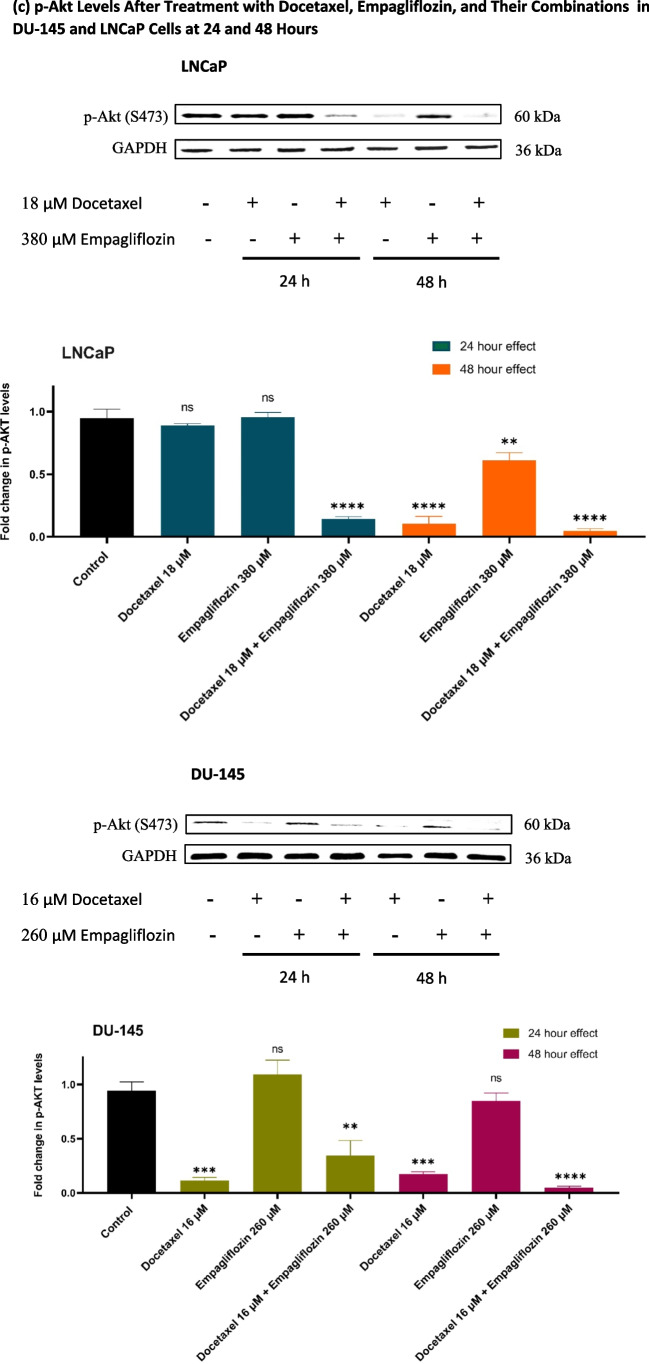
Western blot analyses illustrating the levels of p-AMPKα (**a**), p-p70S6 K (**b**), p-Akt (**c**), and p-PRAS40 (**d**) in LNCaP and DU- 145 prostate cancer cell lines following 24-h and 48-h treatments with 380 μM Empagliflozin, 18 μM Docetaxel, or a combination of both in LNCaP cells, and with 260 μM Empagliflozin, 16 μM Docetaxel, or a combination of both in DU- 145 cells. GAPDH was used as an internal control for quantitative normalization. Data are presented as fold changes relative to untreated control cells, with values expressed as means ± standard deviations (SDs) (*n* = 3). Statistical significance is indicated by * *p* < 0.05, ** *p* < 0.01, *** *p* < 0.001, **** *p* < 0.0001 compared to the control, with"ns"denoting non-significance

The impact of empagliflozin alone and its combination with docetaxel exhibited a time-dependent response, with more pronounced effects observed after 48 h. Compared to the individual treatments with either empagliflozin or docetaxel, the combination demonstrated enhanced efficacy, except for p70S6 K1 in DU- 145 cells, where the effect of docetaxel alone was comparable to that of the combination (Fig. 3b). Moreover, the results revealed distinct p-Akt responses between the DU- 145 and LNCaP cell lines following empagliflozin treatment. At 48 h, LNCaP cells exhibited a significant downregulation of p-Akt levels to approximately 0.4-fold compared to the control, while DU- 145 cells showed no significant change in p-Akt levels with empagliflozin alone (Fig. 3c).

## Discussion

This study presents novel insights into the anticancer potential of empagliflozin, both as a monotherapy and in combination with docetaxel, in LNCaP and DU- 145 prostate cancer cell lines. The observed cytotoxicity and synergistic effects, alongside significant alterations in key molecular pathways, support the hypothesis that empagliflozin activates AMPKα, subsequently inhibiting mTORC1 and its downstream targets, including p70S6 K1 and PRAS40, with cell line-specific variations in p-Akt regulation. These findings contribute to the growing body of evidence indicating that empagliflozin may have therapeutic potential beyond diabetes management, particularly in oncology (Abdelhamid et al. 2022; Karzoon et al. 2025; Xie et al. 2020).

The cytotoxicity data indicate that DU- 145 is more sensitive than LNCaP to both docetaxel (IC_50_: 15.66 μM vs. 17.86 μM) and empagliflozin (IC_50_: 253 μM vs. 378.6 μM), highlighting its greater overall drug sensitivity. This heightened sensitivity of DU- 145, an androgen-independent cell line, to empagliflozin might be related to the presence of SGLT2. Although SGLT2 has been confirmed in prostate cancer patient samples through immunohistochemistry (Scafoglio et al. 2015), its exact expression levels in DU- 145 and LNCaP remain uncertain, which could influence AMPKα activation differences and contribute to the observed outcomes. In DU- 145 cells, empagliflozin increased p-AMPKα levels approximately 22-fold at 48 h, whereas in LNCaP cells, it resulted in a sevenfold increase over the same duration. The variation in response between the two cell lines underscores the need for further research to identify the molecular factors driving this outcome.

The IC_50_ values of docetaxel observed in our study (15.66 μM in DU- 145 and 17.86 μM in LNCaP) align with a previous study by Fujiike et al. (2024), which reported IC_50_ values of 11.06 μM and 14.23 μM in DU- 145 cells. However, the IC_50_ values of docetaxel are consistently reported in the nanomolar range (Attia et al. 2016; Yang et al. 2019). The relatively higher IC_50_ values observed in our study may reflect reduced sensitivity of these cell lines to docetaxel.

The IC_50_ values of empagliflozin in various breast cancer cell lines range from 50 to 320 μM (Eliaa et al. 2020; Karzoon et al. 2025; Nalla and Khairnar 2023). For instance, Nalla and Khairnar (2023) reported an IC_50_ of 320 μM in MCF- 7 cells, aligning with our findings. This variation in IC_50_ values may stem from multiple factors, including differences in glucose concentrations in the culture medium. Our study utilized RPMI- 1640 medium containing 200 mg/dL glucose, mimicking a diabetic environment, whereas glucose levels in tumor tissues are significantly lower, with reported values of 2.1 mg/dL in colon cancer and 7.2 mg/dL in stomach cancer (Hirayama et al. 2009). Methodological differences, such as the choice of assay technique (e.g., xCELLigence, Alamar Blue, or MTT), may also contribute to these discrepancies.

However, clinical dosing in humans yields markedly lower serum concentrations of empagliflozin, ranging from 0.259 to 2.39 μM (Heise et al. 2013), which is substantially below the IC_50_ values observed in vitro. This raises concerns regarding the clinical relevance of empagliflozin’s direct anticancer effects at clinically achievable concentrations. Therefore, further studies replicating the low-glucose tumor microenvironment are essential for evaluating empagliflozin’s anticancer efficacy at clinically relevant doses. Additionally, novel strategies to enhance empagliflozin accumulation in tumors, along with synergistic drug combinations already used in oncology, may improve its therapeutic potential in cancer treatment.

The role of AMPKα in prostate cancer remains complex and inconclusive, necessitating cautious interpretation of its effects. Some researchers argue that AMPK initially acts as a tumor suppressor during the early stages of tumorigenesis but later transitions to a more oncogenic role in advanced stages, contributing to therapy resistance and cancer recurrence (Chhipa et al. 2010, 2011; Frigo et al. 2011; Hardie 2015; Jeon and Hay 2015). Conversely, AMPK activators such as AICAR (aminoimidazole- 4-carboxamide- 1-β-D-ribofuranoside), an AMP mimetic, have demonstrated anti-proliferative effects by reducing cell proliferation in both androgen-sensitive and CRPC models, along with a consistent decrease in de novo fatty acid synthesis (Xiang et al. 2004). In addition, Sahra et al. (2008) demonstrated that metformin effectively inhibits prostate cancer cell proliferation and tumor growth in xenograft models. Similarly, MT 63–78, a specific allosteric activator of AMPK, has been shown to hinder the proliferation of various cancer cells in vitro and suppress the growth of androgen-sensitive tumors in vivo (Zadra et al. 2014). Our findings align with these observations, as we show that empagliflozin and its combination with docetaxel activate AMPKα in a time-dependent manner, with these elevated levels correlating with significantly reduced cell viability in metastatic prostate cancer cell lines, LNCaP and DU145 (Figs. 2 and 3a). These observations suggest that substantial AMPK activation may confer robust anti-proliferative effects, providing potential therapeutic advantages for prostate cancer treatment.

Elevated protein synthesis resulting from the overactivation of mTOR is a hallmark of prostate cancer. These changes are triggered by androgens as well as the dysregulated PTEN/PI3 K/Akt/mTOR signaling pathway, which is disrupted in a substantial proportion of prostate cancer cases (Morgan et al. 2009). Specifically, the tumor suppressor PTEN is affected by deletions and/or mutations in approximately 15–20% of primary prostate cancer cases and over 40–60% of metastatic prostate cancer cases (Leinonen et al. 2013; Lotan et al. 2017; Yoshimoto et al. 2012). Empagliflozin, both alone and in combination with docetaxel, significantly reduced the expression of p-p70S6 K1 in LNCaP and DU145 cell lines. The effects of empagliflozin were closely tied to increased levels of p-AMPKα (Fig. 3a and b). The mechanism by which empagliflozin exerts its action appears to rely on AMPKα activation, which inhibits mTORC1, thereby preventing the phosphorylation and activation of p70S6 K1, a key downstream effector of mTOR signaling. While the individual effects of empagliflozin and docetaxel on p-p70S6 K1 levels in LNCaP cells were modest, their combination resulted in a striking reduction in p70S6 K1 activity by over 98%. This significant reduction suggests a synergistic interplay between the two drugs in LNCaP cells, potentially amplifying their anticancer efficacy.

The contrasting responses of p-Akt to empagliflozin in LNCaP and DU- 145 cells are particularly striking. In LNCaP cells, empagliflozin significantly reduces p-Akt levels, whereas no such downregulation occurs in DU- 145 cells, potentially highlighting differences in androgen receptor (AR) signaling. LNCaP cells, which are AR-sensitive, may experience this effect due to empagliflozin’s modulation of AR activity, leading to a more pronounced suppression of the PI3 K/Akt pathway in AR-dependent contexts. In contrast, DU- 145 cells, being AR-insensitive, do not exhibit this response, suggesting that AR signaling may play a critical role in empagliflozin’s impact on Akt phosphorylation. Nevertheless, this study is unable to offer a conclusive explanation for these disparities, underscoring the necessity for additional research to elucidate the underlying mechanisms. In MCF- 7 breast cancer cells, empagliflozin significantly diminished Akt activity, likely through activation of the FOXO3a-PTEN pathway. In contrast, in LNCaP cells, where PTEN is lost, the reduction in Akt activity was less pronounced, indicating that empagliflozin might inhibit Akt via additional mechanisms independent of the FOXO3a-PTEN pathway (Karzoon et al. 2025; Vlietstra et al. 1998).

The activity of PRAS40 is primarily regulated through phosphorylation. When phosphorylated by Akt at Thr246 and by mTORC1 at Ser183, PRAS40 dissociates from mTORC1, relieving its inhibitory effect on mTORC1 signaling (Nascimento et al. 2010). As a result, the phosphorylation of PRAS40 at Thr246 is closely linked to Akt activity. In empagliflozin-treated LNCaP cells, the reduction in Akt activity led to a corresponding decrease in p-PRAS40 levels. However, in DU145 cells, where empagliflozin did not significantly alter p-Akt levels, p-PRAS40 levels exhibited only a modest change. Notably, the combination treatment with docetaxel enhanced empagliflozin’s effects, given that docetaxel strongly suppressed Akt activity in both LNCaP and DU145 cell.

The combination index values below 1 indicate a synergistic interaction, suggesting that empagliflozin enhances docetaxel’s efficacy. This synergy could enable dose reduction while mitigating resistance—a critical challenge in CRPC (Tannock et al. 2004). The dose-reduction indices exceeding 1 further indicate a beneficial reduction in required drug concentrations, which could minimize toxicity and improve therapeutic windows. This synergy is particularly evident at higher concentrations, such as 300 μM empagliflozin with 20 μM docetaxel in LNCaP cells, highlighting a dose-dependent synergistic mechanism.

This study provides novel insights into the anticancer effects of empagliflozin, but several limitations remain. First, all experiments were conducted in vitro, which does not fully replicate the tumor microenvironment. Future studies with xenograft or genetically engineered mouse models are essential to confirm these findings in a more physiologically relevant setting. Second, while cytotoxicity is a key measure of anticancer activity, additional functional assays, such as colony formation, migration, and invasion assays, could provide further insights into the effects of the empagliflozin-docetaxel combination on tumor progression. Future studies incorporating these analyses will help validate and expand on our findings. Third, while our data suggest empagliflozin acts via AMPKα activation and mTORC1 inhibition, other mechanisms, such as metabolic stress or mitochondrial dysfunction, remain unexplored. Investigating these broader effects could provide a more comprehensive understanding. Fourth, the differences in Akt phosphorylation between LNCaP and DU- 145 cells highlight the need for further studies on AR signaling and other regulatory pathways. Additionally, confirming SGLT2 expression in these cell lines will clarify whether empagliflozin directly targets this transporter in prostate cancer. Finally, the observed synergy between empagliflozin and docetaxel warrants deeper investigation to determine whether it enhances treatment efficacy via apoptosis induction, cell cycle regulation, or resistance modulation.

## Conclusion

This study demonstrates that empagliflozin reduces prostate cancer cell viability through AMPKα activation and inhibition of p70S6 K1 and PRAS40, with differential effects on Akt phosphorylation in LNCaP and DU- 145 cells. Its combination with docetaxel exhibits synergistic anticancer effects. This synergy suggests that empagliflozin may enhance docetaxel efficacy, potentially enabling dose reductions and improving treatment outcomes. These findings support the potential repurposing of empagliflozin in oncology, warranting further investigation through additional in vivo studies and clinical trials to assess their translational relevance.

## Supplementary Information

Below is the link to the electronic supplementary material.

Supplementary file1 (PDF 282 KB)

## Data Availability

All source data for this work (or generated in this study) are available upon reasonable request.

## Abbreviations

AMPKα: AMP-Activated Protein Kinase α
AR: Androgen Receptor
CI: Combination Index
CRPC: Castration-Resistant Prostate Cancer
DRI: Dose Reduction Index
mTORC1: Mammalian Target of Rapamycin Complex 1
PRAS40: Akt Substrate of 40 kDa
PTEN: Phosphatase and Tensin Homolog
p70S6 K1: P70-S6 Kinase 1
SGLT2: Sodium-Glucose Cotransporter 2
