# Supplementary figures and images for: Effects of empagliflozin and its combination with docetaxel on LNCaP and DU- 145 prostate cancer cell lines: cytotoxicity and molecular pathway analysis
Source: Naunyn Schmiedebergs Arch Pharmacol. 2025 Apr 14;398(10):13819–31. doi: 10.1007/s00210-025-04132-9 (PMC12511237; doi:10.1007/s00210-025-04132-9)

DU-145 Cell Line

p-Akt

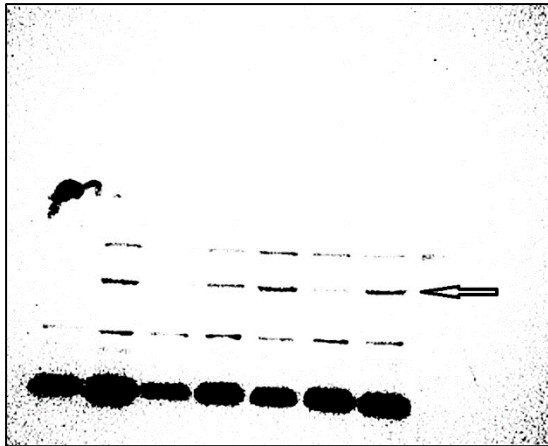

p-AMPK $\alpha$

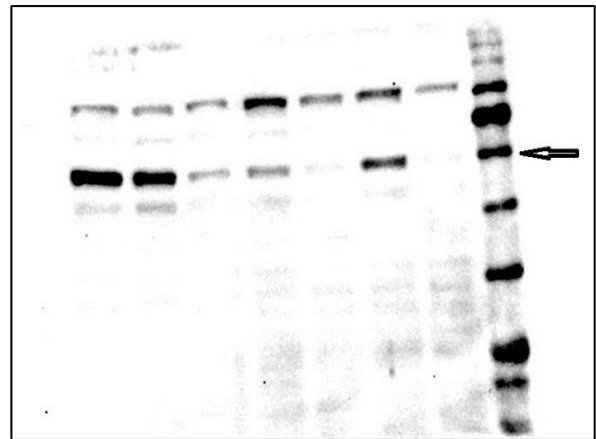

GAPDH

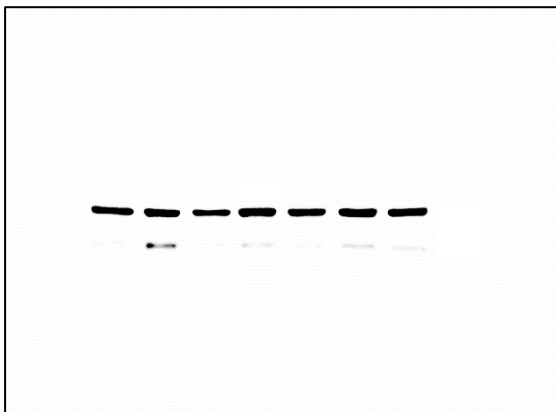

p-p70S6k1

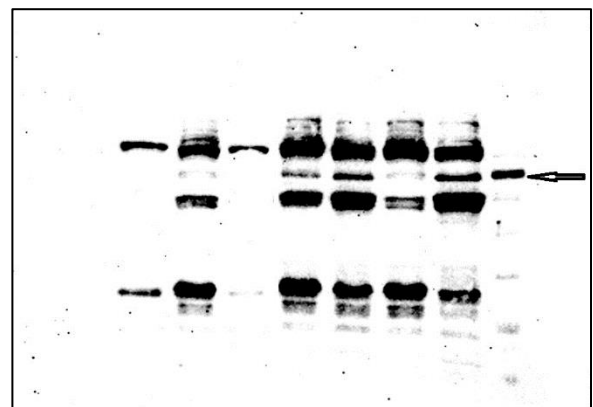

p-PRAS40

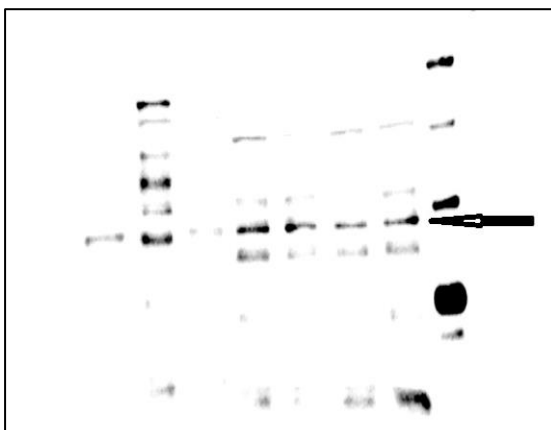

LnCAP Cell Line

GAPDH

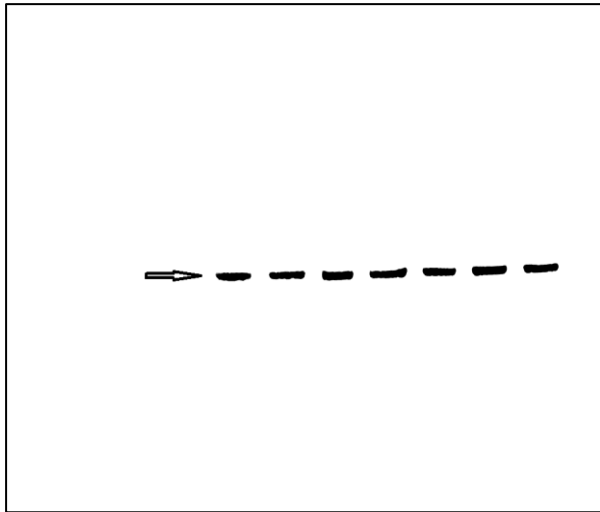

p-p70S6K1

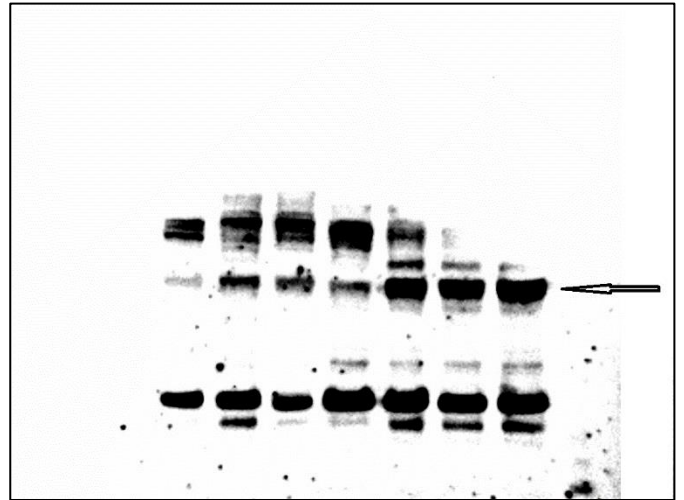

p-Akt

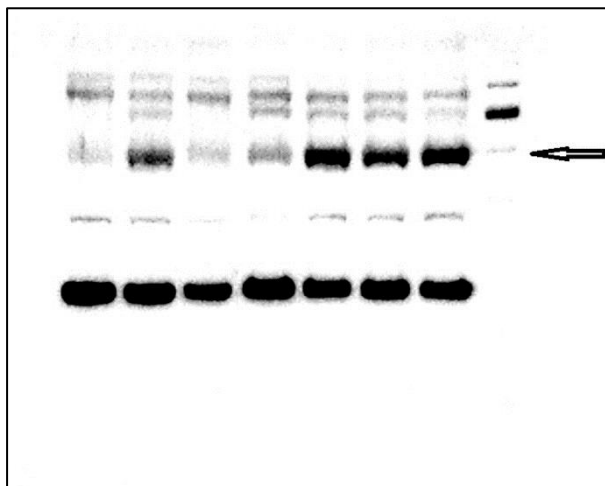

p-PRAS40

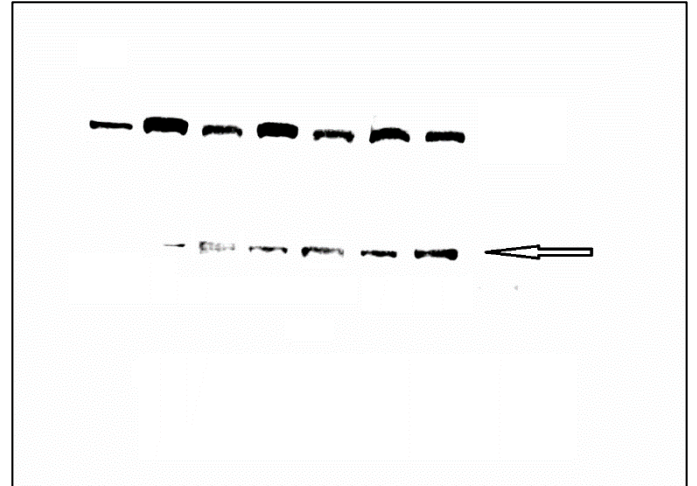

p-AMPK $\alpha$

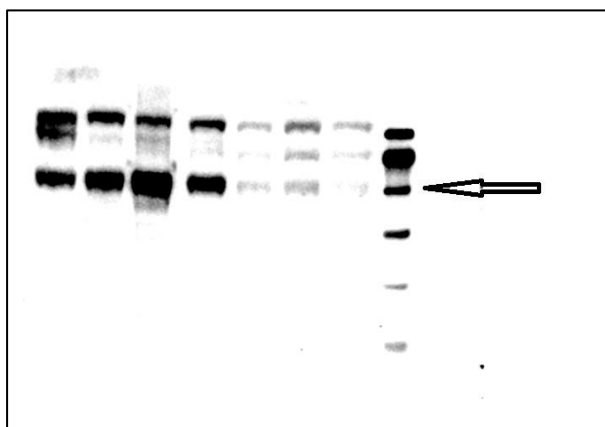

Supplement: Supplementary file 1 — Supplementary file1 (PDF 282 KB) [file 210_2025_4132_MOESM1_ESM.pdf]
